# A method for comparing intra-tumoural radioactivity uptake heterogeneity in preclinical positron emission tomography studies

**DOI:** 10.1186/s40658-015-0124-1

**Published:** 2015-09-08

**Authors:** Jonas Grafström, Hanna-Stina Ahlzén, Sharon Stone-Elander

**Affiliations:** Department of Clinical Neuroscience, Karolinska Institutet, SE-17176 Stockholm, Sweden; Division of Biochemistry, Department of Medical Biochemistry and Biophysics, Karolinska Institutet, SE-17177 Stockholm, Sweden; PET Radiochemistry, Neuroradiology Department, Karolinska University Hospital, SE-17176 Stockholm, Sweden

**Keywords:** Uptake heterogeneity, Small animal imaging, Positron emission tomography, Textural analysis, Xenografts

## Abstract

**Background:**

Non-uniformity influences the interpretation of nuclear medicine based images and consequently their use in treatment planning and monitoring. However, no standardised method for evaluating and ranking heterogeneity exists. Here, we have developed a general algorithm that provides a ranking and a visualisation of the heterogeneity in small animal positron emission tomography (PET) images.

**Methods:**

The code of the algorithm was written using the Matrix Laboratory software (MATLAB). Parameters known to influence the heterogeneity (distances between deviating peaks, gradients and size compensations) were incorporated into the algorithm. All data matrices were mathematically constructed in the same format with the aim of maintaining overview and control. Histograms visualising the spread and frequency of contributions to the heterogeneity were also generated. The construction of the algorithm was tested using mathematically generated matrices and by varying post-processing parameters. It was subsequently applied in comparisons of radiotracer uptake in preclinical images in human head and neck carcinoma and endothelial and ovarian carcinoma xenografts.

**Results:**

Using the developed algorithm, entire tissue volumes could be assessed and gradients could be handled in an indirect manner. Similar-sized volumes could be compared without modifying the algorithm. Analyses of the distribution of different tracers gave results that were generally in accordance with single plane preclinical images, indicating that it could appropriately handle comparisons of targeting *vs.* non-targeting tracers and also for different target levels. Altering the reconstruction algorithm, pixel size, tumour ROI volumes and lower cut-off limits affected the calculated heterogeneity factors in expected directions but did not reverse conclusions about which tumour was more or less heterogeneous.

**Conclusions:**

The algorithm constructed is an objective and potentially user-friendly tool for one-to-one comparisons of heterogeneity in whole similar-sized tumour volumes in PET imaging.

## Background

In nuclear medicine imaging, non-uniformity or heterogeneity in radiotracer uptake in tissues is visually perceived as areas of high as well as low uptake. Random and systematic factors such as the Poisson distribution of the radioactive decay and noise in addition to the processing parameters used to generate the images [1–6] can contribute to image heterogeneity as well as variations in uptake due to the non-homogenous features of the tissues being analysed (e.g. [7–11]). Heterogeneity in tumour imaging is hypothesised to be a potentially important indicator of variations in the underlying biology such as differences in structural features, cellular density, metabolism, growth rate, receptor populations, vascularisation, hypoxia, impaired lymphatics or the varying effects on these that therapy may induce throughout the lesion [12–19]. Recognising and interpreting differences in uptake patterns can therefore be important for decisions about patient prognosis and for recommendations about specific therapeutic strategies (e.g. [20–25]). An increasing number of strategies for analysing image heterogeneity have been published over the last few years (e.g. [26–32]). These methods are often compared with the conventional analyses performed visually by imaging experts. As of yet, there is no standardised method available for analysing heterogeneity.

Methods developed for oncological imaging applications are often performed in preclinical disease models before implementation in human studies. Experimental tumour models grow much more rapidly than in humans and morphological and functional properties may therefore vary on a time scale of days to weeks instead of months to years. Issues that may be present in studies of human tissues may become critical very quickly in preclinical studies. It can also be very difficult to evaluate non-uniformity visually since the dimensions of tumours are only on the order of a few millimetres in these rodent models of human tumours. Although the fact that heterogeneity has an impact on quantifications performed has been recognised in many studies (e.g. [14, 15, 33]), methods for estimating specifically the intra-tumoural uniformity in preclinical tumours are not yet, to our knowledge, available.

Currently, there is no general consensus about exactly what constitutes heterogeneity in an image, the factors affecting it nor how it should be ranked or estimated (see e.g. [6]). Most methods developed so far for analysing clinical images have used some sort of texture analysis. In this paper, we develop and examine the application of a texture-based algorithm to assess radioactivity uptake heterogeneity in planes and in the sum of planes through preclinical tumour xenografts studied with small animal positron emission tomography (PET). The algorithm identifies and subsequently isolates deviations from the mean uptake. The absolute values of these deviations are then computed (hereafter denoted “peaks”). The mean peak intensity of every pair of peaks is subsequently divided by the distance between these peaks, the distributions of these deviations are plotted in histograms and a heterogeneity factor (HF) is calculated. The method is applied to different types of comparisons typically performed in preclinical small animal PET investigations of tumour models: comparing (I) the uptake of size-matched targeting *vs.* non-targeting radiotracers in the same tumour, (II) the uptake of the same tracer in tumours with different expression levels of the target and (III) the uptake of different tracers targeting a tumour by different mechanisms. This analysis only focuses on the spatial heterogeneity of the uptake intensities. The implications of differences observed for the underlying pathophysiology will, of course, require many more complementary investigations. Efforts have been made to design this algorithm so it is easy to follow, so it performs a complete rather than a partial analysis of the tumour and so that it is very general, i.e. no code modifications are required for different situations.

## Methods

### General

The images retroactively analysed with the algorithm developed here have been acquired and reported in previously published studies [34, 35] in which the experimental details can be found. In brief, the animals analysed here were severe combined immunodeficiency (SCID) mice carrying subcutaneous tumour xenografts of head and neck (FaDu) and epidermal (A431) and ovarian (SKOV-3) carcinoma cell lines. Experiments were performed in accordance with national legislation on laboratory animals’ protection and were approved by the local ethics committee for animal research (Stockholm north ethical committee (animal research)). All animal handling was performed by the same individual. For radioligands, the methyl-^11^C-radiolabelled Annexin A5, [methyl-^11^C]-His_6_-AnxA5-ST-CH_3_, hereafter denoted AnxA5 (~38 kDa), mutated-thioredoxin-green fluorescence protein [methyl-^11^C]-His_6_-mTrx-GFP-ST-CH_3_, hereafter denoted mTrx-GFP (~40 kDa) and the Affibody™ Z_HER2:342_ ([methyl-^11^C]-Z_HER2:342_-ST-CH_3_) hereafter denoted Z_HER2:342_ (~7 kDa) proteins had been expressed with a C-terminus selenocysteine tag (ST) and site specifically labelled with a positron-emitting carbon-11 (^11^C) (*t*_1/2_ ≈ 20 min) methyl group (CH_3_). The widely employed 2-deoxy-2-[^18^F]fluoro-D-glucose [^18^F]FDG (~0.18 kDa) also used here was obtained in an aliquot from batches made daily for clinical PET at the Karolinska University Hospital.

### Data acquisition and handling

The PET camera used was the microPET Focus 120 (Concorde, Siemens), whose performance has been previously evaluated [36]. The field-of-view (FOV) is 7.6 × 10 cm for axial to transaxial dimensions and the resolution of the machine centrally in the FOV (CFOV) is about 1.2 mm. Data were continuously sampled for 1 h in list mode, corrected for dead time, randoms and physical decay and histogrammed for this study as follows: 3 s × 20 frames, 150 s × 8 frames and 293 s × 8 frames. Subsequently, they were reconstructed using ordered subset estimation maximum in 2 dimensions (OSEM2D) in order to increase the spatial resolution. This was performed with a picture size of 512 × 512 pixels, 4 iterations and 16 subsets. Since the dimensions of all tumours studied here were more than four times the resolution of the CFOV, partial volume effects (PVEs) due to the size of the lesion were minimised [37]. For all imaging situations, the same hardware and software were used and hence the same image bit depth was employed. The software used to define and calculate the radioactivity within volumes of interest (VOIs) was the Inveon Research Workplace (IRW) developed by Siemens. The VOI was manually drawn in coronal, sagittal and transaxial planes in the summed images for each separate tumour. Radioactivity concentrations were calculated automatically by calibration against a phantom with a known concentration of radioactivity.

The uptake of radiotracers in the tumours was calculated as a standard uptake value (SUV) [38] in which the regional activity is related to the total injected dose and normalised to standardise for between-individual comparisons. The SUV is defined as

1 $$ \mathrm{S}\mathrm{U}\mathrm{V}=\frac{\mathrm{Radiotracer}\;\mathrm{concentration}}{\mathrm{Injected}\;\mathrm{activity}/\mathrm{normalisation}\ \mathrm{factor}} $$

The normalisation factor can be related to body surface area, lean body mass and body weight. Here, the SUVs were normalised to body weight. SUVs were computed for every reconstructed time frame. For this study of uptake heterogeneity, images summed over the last 30 min were used. In future analyses, heterogeneity in the specific binding of the radiotracers might be further analysed by using other calculated macroparameters such as the binding potential or distribution volumes.

### Algorithm for analysis of heterogeneity

**Fig. 1:**
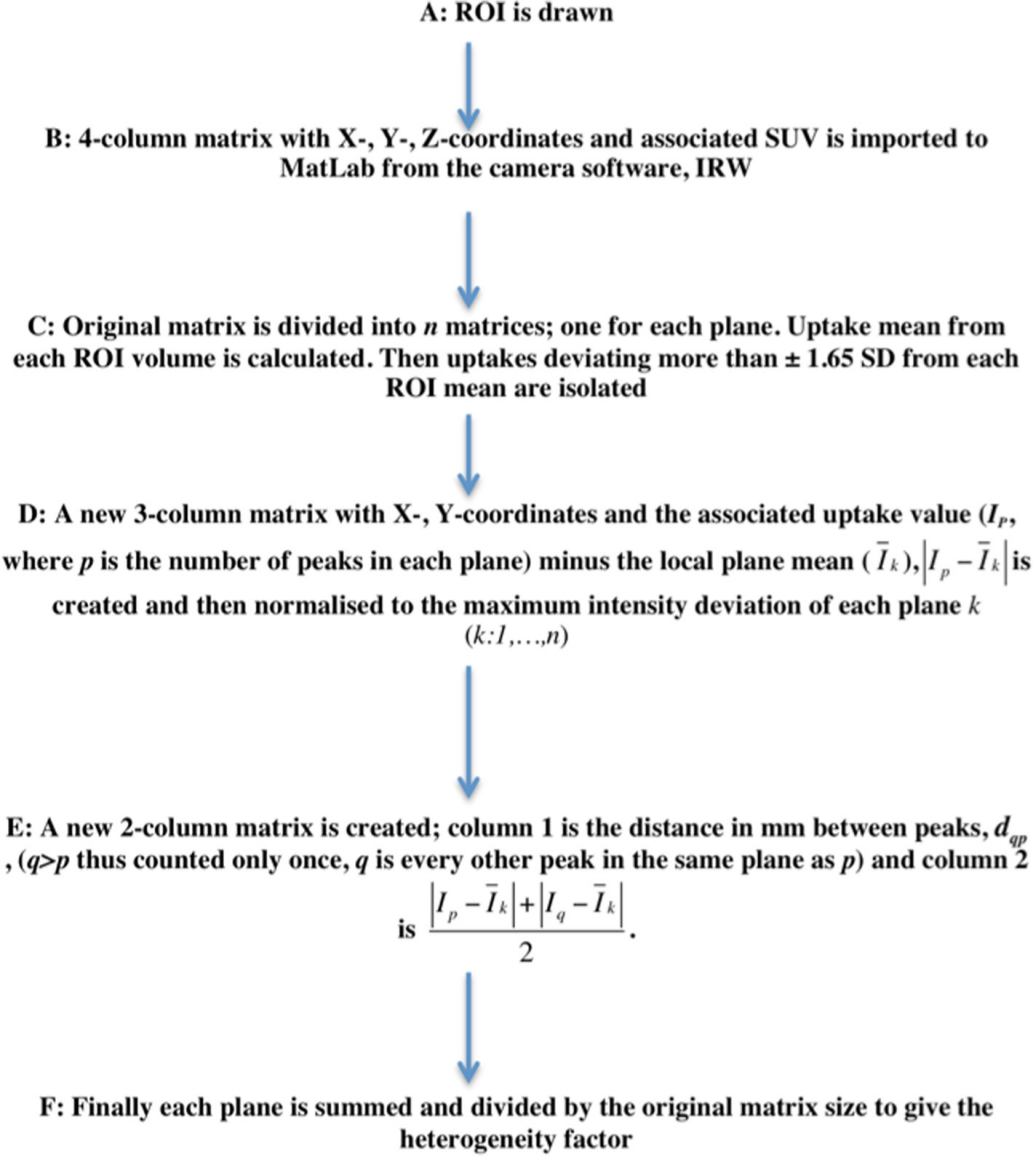
The outline of the algorithm

Since the uptake heterogeneity is defined as the deviations from a mean uptake per unit distance, clustered deviations, i.e. those in close proximity to each other, should have a larger weight or impact on any ranking of heterogeneity than deviations that are further apart. Therefore, mean uptake deviations are normalised to the associated distances between them. The steps in the algorithm for assessing heterogeneity are presented schematically in Fig. 1. Each step is explained in more detail below.

#### VOI definition

The method for drawing the VOI is very important in the analysis of heterogeneity. The method used needs to be pre-established and consistently followed throughout an analysis. Some type of thresholding to delineate “viable” tumour is often used to objectively include only areas with uptake above a certain level. However, basing the VOI on only the high uptake areas would exclude “colder” areas that could have a considerable impact on the heterogeneity [39]. Therefore, in this analysis, the so-called primary VOI was instead drawn as a sphere or ellipsoid that completely covered the tumour when considerable uptake of radioactivity was observed, in this case in the time frames after 30 min. This primary VOI was only used to make sure that all the tumour tissues were included but was not itself used in the calculations. Instead, a second VOI, based on tumour dimensions measured postmortem, was then drawn manually within the primary VOI for each separate tumour and fitted to the tumour dimensions in the sagittal, coronal and transaxial planes of the images. Thresholding was not performed at this step but rather the entire “secondary” VOI matrix was used in the calculations of heterogeneity.

#### First VOI data post-processing

Discrete volume elements (voxels) make up the image VOI. Every voxel is associated with four values: the three spatial coordinates; the *X-*, *Y-* and *Z*-positions, and the voxel intensity (measured here as SUVs) and these are arranged as a matrix with four columns. This VOI data is subsequently exported from IRW to MATLAB (version R2011a) in which the heterogeneity algorithm had been constructed and all subsequent steps are performed.

#### Subdivision of one four-column matrix into *n* three-column matrices

The original, VOI defining matrix is first subdivided into *n* matrices, one for each plane, in order to evaluate each plane separately. To locate the uptake values that compose the deviations from the local matrix-mean or the heterogeneity, thresholding was applied to isolate intensities above and below a pre-set threshold value—here ±1.65 standard deviations (for normally distributed measurements, about 68 % fall within 1 SD of the mean, about 98 % fall within 2 SD of the mean and about 90 % fall within 1.65 SD of the mean), along with their positional coordinates.

To determine the type of distribution, a quantile-quantile (QQ) plot, (MATLAB), which plots a theoretical normal distribution together with the current data, was performed on the pixel values. The data were considered to be consistent with a normal distribution.

#### Recreation of a cell array with individual intensities related to the mean of the associated plane

The deviating uptakes found in the previous step are now used to create a new three-column matrix, with the *X*- and *Y*-positions and the absolute values of the intensity deviations subtracted from the mean intensity of the associated plane (where the mean *I* is the *I-*bar in Eq. 2). Each individual |*I*_*p*_ − *Ī*_*k*_| is normalised to the maximum deviation locally in order to compensate for individual uptake deviations.

Each individual heterogeneity, the mean intensity deviation per distance (in millimetre), here is defined as:

2 $$ \left(\frac{\left|{I}_p-{\overline{I}}_k\right|+\left|{I}_q-{\overline{I}}_k\right|}{2}\right)/ \max\;\left(\frac{\left|{I}_p-{\overline{I}}_k\right|+\left|{I}_q-{\overline{I}}_k\right|\;}{2}\right)\cdot \frac{1}{d_{pq}} $$

Each plane is, as mentioned above, evaluated separately. This means that we look at the distance *X*_*1*_,*Y*_*1*_ to *X*_*2*_,*Y*_*2*_ only for a specific *Z.* The reasons for this are twofold. First, heterogeneity can vary considerably from one plane to another. To be generally useful for analysing tumours of different sizes, evaluating each plane separately allows a certain control of which factors are influencing changes in the HF when more planes are added. The second reason is so that the impact of the gradient or slope (i.e. the steepness of the incline or decline) can be addressed. If we have a deviation with a steep slope, the contribution must be different than when the deviation has a gradual, lower slope, even if the peak value is the same.

Thus, the heterogeneity *H* for each plane *k* (*k:1,…,n* for a total of *n* planes) and each peak *p* (*p:1,..,m* for a total of *m* peaks) is (where p > q)

3 $$ H(k)={\displaystyle \sum_1^m\left(\frac{\left|{I}_p-{\overline{I}}_k\right|+\left|{I}_q-{\overline{I}}_k\right|}{2}/ \max \left(\frac{\left|{I}_p-{\overline{I}}_k\right|+\left|{I}_q-{\overline{I}}_k\right|}{2}\right)\cdot \frac{1}{d_{pq}}\right)} $$

When the slope is very gradual, the contribution to the mean will have a more pronounced effect than that of a steep slope. This is why the method by which a deviating peak is accepted or rejected will have an impact on how the gradient is treated or, consequently, how the gradient impacts the algorithm. In calculating the deviations from the mean, a peak is either accepted or rejected by the use of thresholding. When each plane is evaluated separately, the mean from each plane is used instead of a global mean for the entire tumour volume. When a global mean is used, only two possible alternatives, i.e. “yes it should be included” and “no, it should not be included”, are possible. When the thresholding is instead performed for a local mean, i.e. for each separate plane, there will still be only two possible alternatives, but the acceptance or rejection of peaks occurs many more times. Thus, more alternatives are introduced for managing the influence of a gradient or the spatial surrounding of a deviating peak.

#### Creation of a simplified cell array based on pair-wise means and their respective distances

**Fig. 2:**
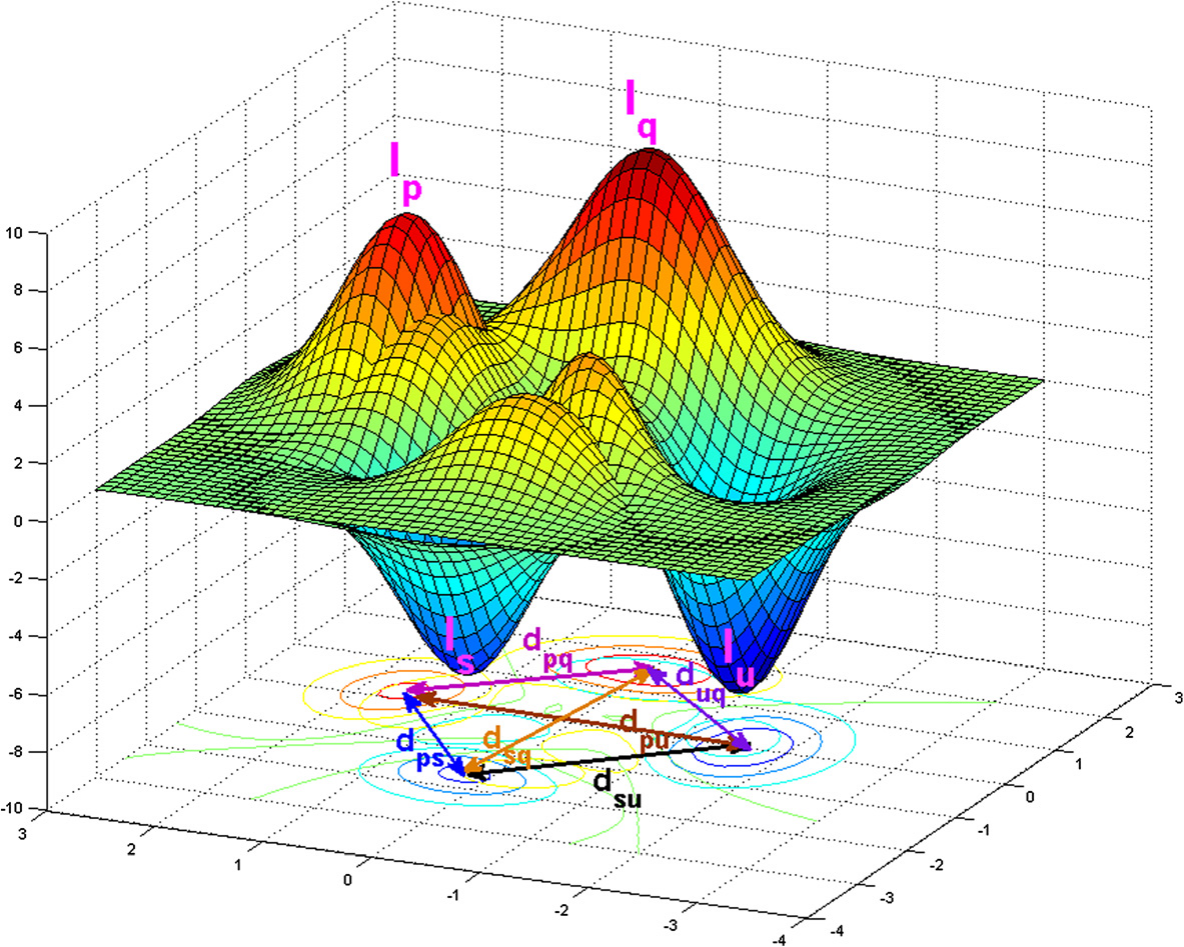
Visualising the results of the application of the algorithm. The heterogeneity contribution for one plane (*k*) would be calculated as $H(k)={\displaystyle \sum_1^m\left(\frac{\left|{I}_p-{\overline{I}}_k\right|+\left|{I}_q-{\overline{I}}_k\right|}{2}/ \max\;\left(\frac{\left|{I}_p-{\overline{I}}_k\right|+\left|{I}_q-{\overline{I}}_k\right|\;}{2}\right)\cdot \frac{1}{d_{pq}}\right)}$

Each pair of intensity deviations is now recalculated as a mean (Eq. 2) and the 2-D Euclidean distance between every intensity deviation in the matrix created in the previous step is calculated (Fig. 2). When the distances (*d*_*pq*_) are less than the resolution of the camera at CFOV (1.2 mm) or larger than the minimum tumour dimension (here 4 mm), those paired intensity deviations are omitted. Omitting distances that are too small also avoids including voxels that might be of the same peak. Thus, a two-column matrix is formed in which the first column contains the distances and the second column contains the means of the two actual intensity deviations from the associated mean.

#### Calculation of the heterogeneity factor for the whole VOI

When all planes have been evaluated separately, the separate HF for each plane is summed. To compensate for VOI size, the resulting sum of intensities is divided by the original matrix length, e.g. the number of voxels in the VOI. This then gives the heterogeneity factor.

The distributions of the varying contributions to the HF for the entire tumour volume were calculated and displayed as histograms with 1000 bins for each analysis. Since the distances are limited to 1.2–4 mm, the values of the HF are distributed from 0 to 1/1.2 ≈ 0.83 (we have allowed the *X*-axis to continue to 1 for aesthetic reasons). The histogram is formed in such a way that the values from Eq. 2 are distributed along the *X*-axis in 1000 bins that are all equally spaced and they all have the same width. These histograms more readily visualise the frequency of deviations, the groups of deviations and the breadth of the spectrum of deviations. Also, it can be seen for which mean intensity deviation the contributions to the HF occur.

Thus, the heterogeneity factor *HF* is

4 $$ HF=\frac{{\displaystyle \sum_1^nH(k)}}{length(ROI)} $$

### Test validations of the heterogeneity algorithm

During the development of the heterogeneity algorithm, its ability to correctly handle different uniformity patterns was validated using simulated four-column matrices similar in set-up to those generated by a VOI but controlled. A typical length of a VOI-matrix was about 3000 rows while these “test”-matrices were only 18 rows long. Furthermore, the “test”-matrices were only composed of integers while a VOI-matrix is composed of fractional numbers and all numbers are therefore unique. The “test-matrices” were engineered so that one particular property (e.g. low, intermediate and large intensity fluctuations and varying types of intensity gradients) was amplified so the influence of this feature could be tested.

Once the algorithm was found to handle the test matrices appropriately, it was applied in the analyses of three types of comparisons typically made in preclinical imaging. Finally, in the last comparison, the effects of using a different reconstruction algorithm, pixel sizes, tumour VOI volumes and the lower cut-off limit, which is related to the resolution (here the CFOV) on the calculated heterogeneity factor for the uptakes of [^18^F]FDG and AnxA5 were examined in four FaDu tumour-bearing animals. Furthermore, the validity of the HF calculated for the whole VOI was tested by permuting the planes in the VOI to be along the *X-* or *Y-*axis instead of the *Z-*axis.

## Results

In the course of developing the heterogeneity algorithm, the test matrices were mathematically designed so they could be used to confirm the structure and performance of the algorithm. In all validations prior to its application on test situations, the algorithm was successively modified until the hypothesised outcome of the effect of the particular property being tested was achieved. The algorithm can in principle be applied to analyse heterogeneity in any tissue. Preclinical tumour models, once they have established and begun to grow, may change rapidly in both size as well as in heterogeneity. Large changes over time in the tumour size and the underlying biochemistry will therefore definitely affect heterogeneity. Here, we have instead used the algorithm to analyse potentially more modest differences in comparisons commonly made when imaging preclinical tumours of similar size and/or similar stages of development.

### Heterogeneity differences when using same-size but targeting vs. non-targeting tracers (AnxA5 and mTrx-GFP) in a FaDu xenograft

Comparing different radiotracer investigations may often be desirable in order to probe different features of the tissue being targeted. In this example, we examined how the heterogeneity algorithm would describe the uptake of two labelled medium-sized proteins in the same tumour xenograft, i.e. same day, same animal, same tumour, same size but different tracers. The first tracer was based on the 36-kDa protein AnxA5 that binds with phosphatidylserine that is exposed during cell death. The second tracer was a size-matched, non-targeting control protein, mTrx-GFP. This tracer was used previously [34] to estimate the degree of passive uptake and retention effects [40] on the total AnxA5 uptake, though differences in local patterns of uptake of the two tracers were not specifically examined.

**Fig. 3:**
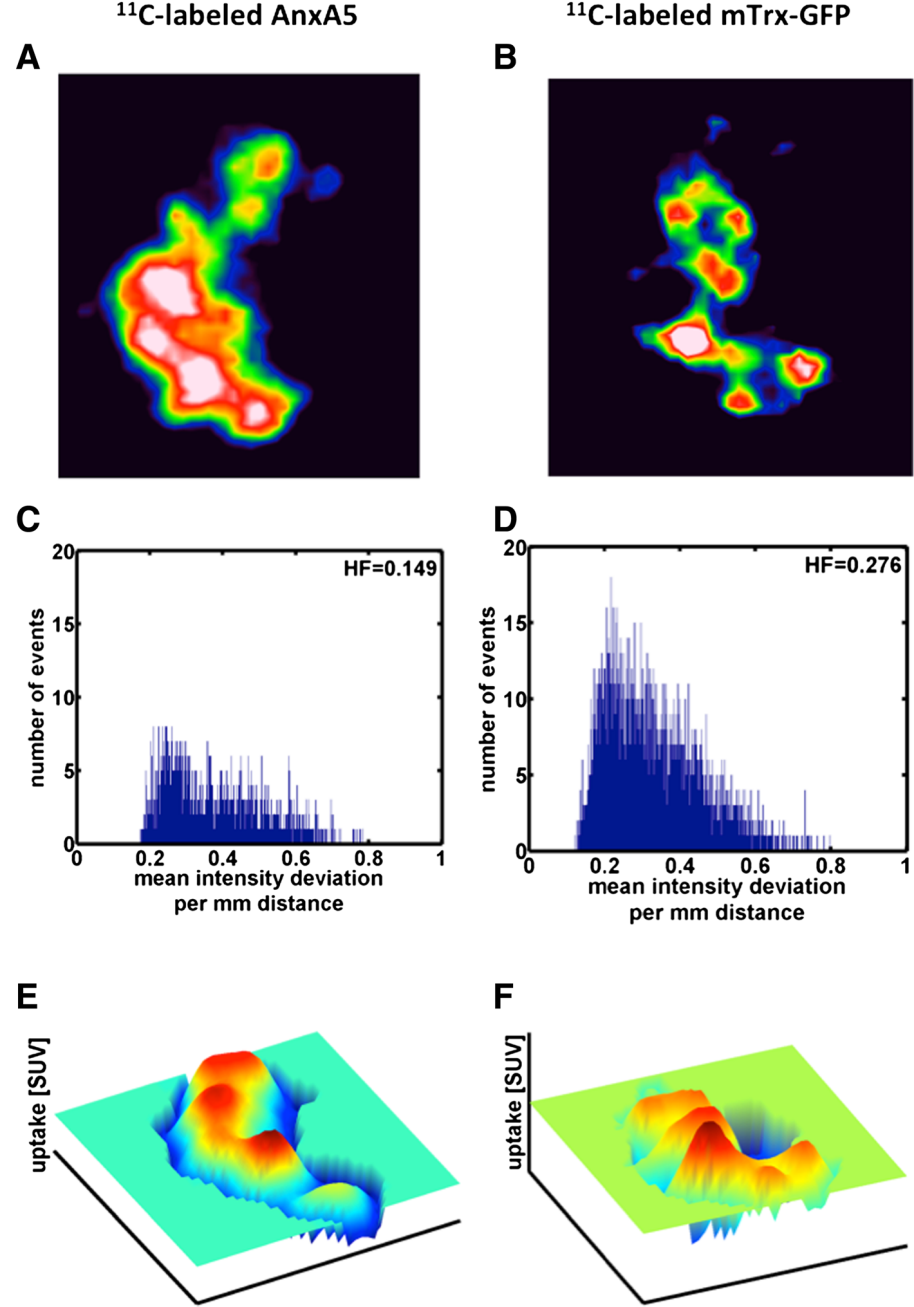
PET transaxial images (**a**, **b**, the colour scales are the same), histograms (**c**, **d**) of the heterogeneity contributions (the mean intensity deviation per distance calculated according to Eq. 2) and surface plots (**e**, **f**) of the uptake of AnxA5 and mTrx-GFP in a FaDu xenograft. The imaging was performed in the same animal >2 h apart on the same day. In **e** and **f**, the *X*- and *Y*-axes represent spatial dimensions and the *Z*-axis is the mean tracer uptake (SUV_mean_)

Both the maximum uptake (SUV_max_) and the mean uptake (SUV_mean_) for AnxA5 were higher than for mTrx-GFP (2.67 and 1.76 *vs.* 1.95 and 1.13, respectively). In the two images through one plane (Fig. 3a, b or, alternatively, Fig. 3e, f) the uptake of mTrx-GFP appeared varied and patchy while that of AnxA5 was more uniformly distributed throughout the tumour. The uptake of mTrx-GFP showed more deviations from the mean (i.e. more frequent and larger peak-to-valley variations). Applying the algorithm, the HF for mTrx-GFP was calculated to be about 85 % higher than that for AnxA5. The plots of the histograms of the heterogeneity contributions in the whole tumour volume (Fig. 3c, d) illustrate that there are more deviations from the mean or a larger frequency in the deviations for mTrx-GFP compared to AnxA5.

### Heterogeneity differences when using the same tracer (Z_HER2:342_) in different tumour models with differing target expression levels

One commonly used method for testing the specificity of a radiotracer’s targeting ability is to determine the radiotracer uptake in different tumour models with different levels of target expression. In this case, we examined how the heterogeneity analysis handled markedly different radiotracer uptake levels. As an example, we examined the previously reported [35] substantially different uptakes of the 7-kDa HER2-targeting protein Z_HER2:342_ in SKOV-3 and A431 xenografts, i.e. different animal, different tumours and target expressions, but the same tracer. The differences in uptake are consistent with the high and intermediate expressions of HER2.

**Fig. 4:**
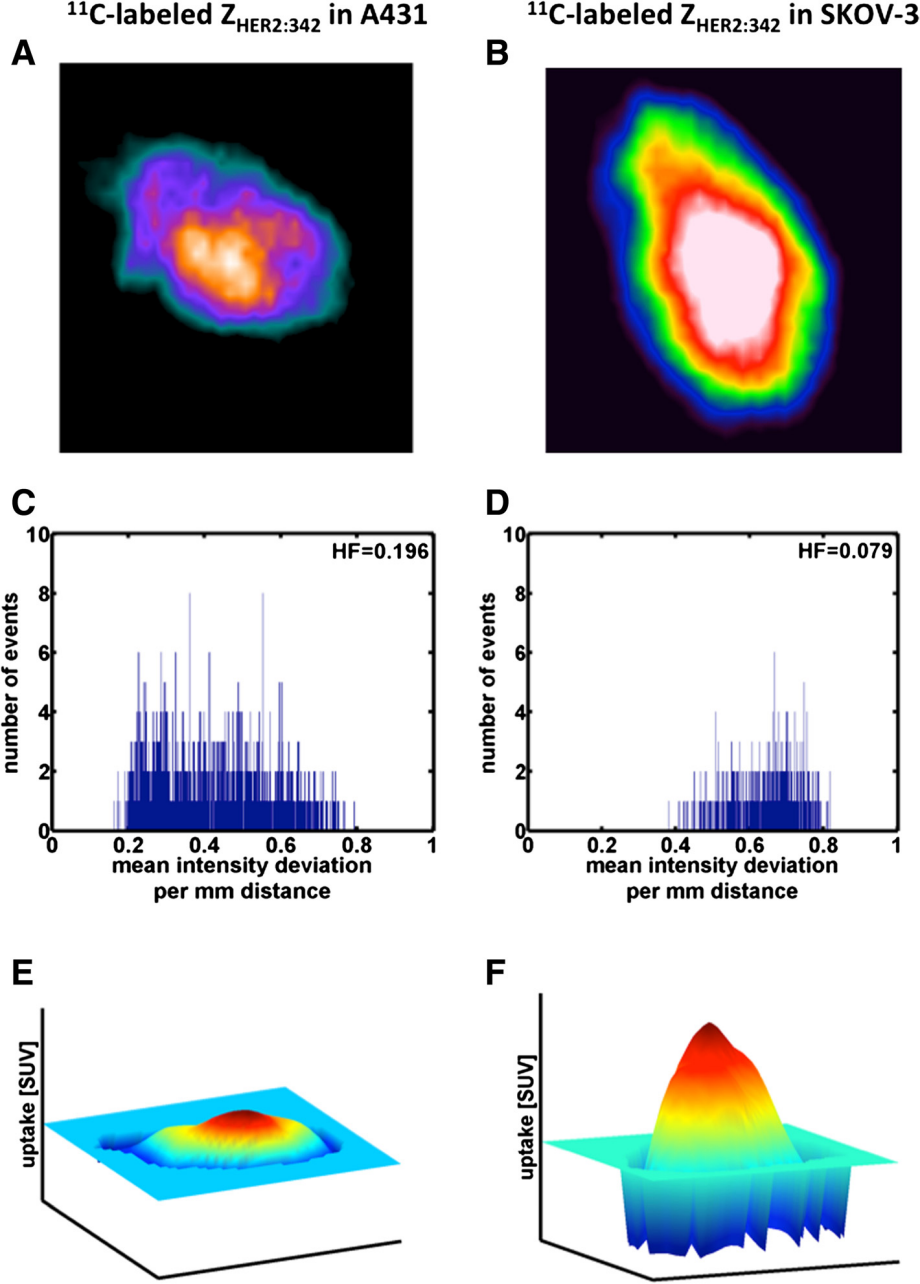
PET transaxial images (**a**, **b**, please note that the scales in these two images are not the same, which is additionally emphasised by using different colour schemes), histograms of the heterogeneity contributions (the mean intensity deviation per distance calculated according to Eq. 2) (**c**, **d**) and surface plots (**e**, **f**) in the A431 and SKOV-3 xenografts (with high and intermediate expressions of HER2 targets, respectively) imaged with the tracer ^11^C-labelled Z_HER2:342_. These data are from two animals bearing xenografts of similar dimensions. In **e** and **f**, the *X*- and *Y*-axes represent spatial dimensions and the *Z*-axis is the uptake in SUV_mean_

The uptake in A431 was low throughout the xenograft, as indicated by the surface plot in Fig. 4e. The uptake was also quite heterogeneous, as was suggested by the transaxial image (Fig. 4a) and supported by the very large number and the broader spectrum of mean intensity deviations in the histogram (Fig. 4c). The HF for uptake in A431 was approximately 2.5 times larger than for SKOV-3. The normalisation applied here compensated adequately for the influence of the much higher uptake in SKOV-3 (Fig. 4f *vs.*4e). Higher uptakes will give a larger |*I*_*p*_ − *Ī*_*k*_|, which, without normalisation, will otherwise automatically lead to a higher HF.

### Heterogeneity differences when using different size radiotracers, [^18^F]FDG and AnxA5, accumulating by different mechanisms in the same tumour

In this analysis, we compared the heterogeneity of the uptake of two tracers, [^18^F]FDG and AnxA5, in the same tumour, i.e. same day, same animal, same tumour, but different tracers of different size and retention mechanisms. These tracers differ in many aspects. For instance, [^18^F]FDG is about 0.5 % the size of AnxA5 and it accumulates primarily in proportion to the metabolic demand of the tissue. The uptake and retention of AnxA5, on the other hand, is affected by its larger size, by the vascular leakiness and lymphatic drainage of the tissue being examined and by the expression of its target phosphatidylserine during on-going cell death in that tissue. The higher positron energy of ^11^C leading to poorer imaging resolution may also influence comparisons of the tracer uptakes.

**Fig. 5:**
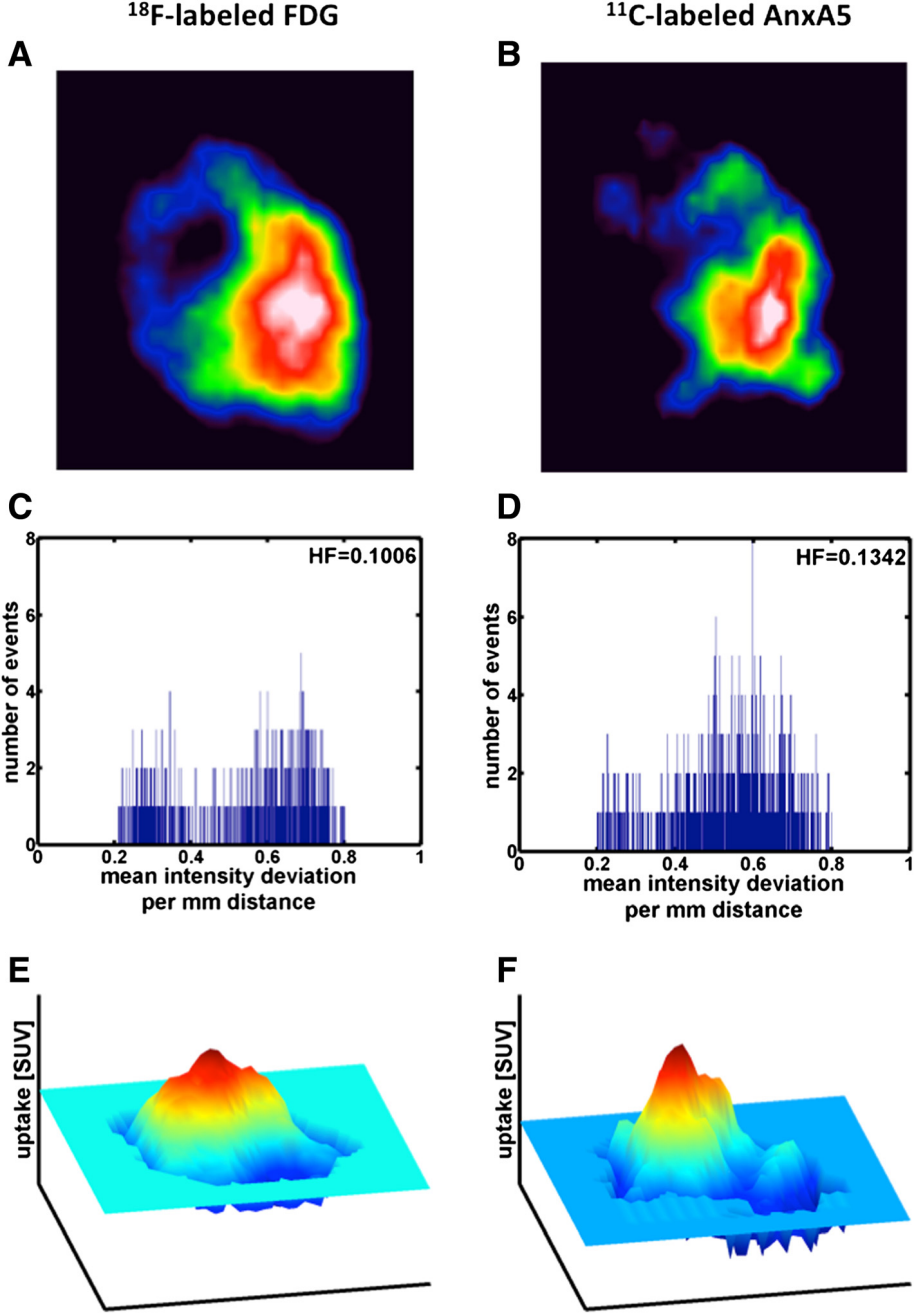
PET transaxial images (**a**, **b**, the colour scales are the same), histograms of the heterogeneity contributions (the mean intensity deviation per distance calculated according to Eq. 2) (**c**, **d**) and surface plots (**e**, **f**) of the uptake of [^18^F]FDG and ^11^C-labelled AnxA5 in a FaDu xenograft. The imaging was performed in the same animal >2 h apart on the same day. In **e** and **f**, the *X*- and *Y*-axes represent spatial dimensions and the *Z*-axis is the tracer uptake in SUV_mean_

The tumour images in Fig. 5a, b indicate that the uptakes of the two tracers have similar patterns but with some textural differences. The HF for [^18^F]FDG is about 25 % less than the HF for AnxA5. The histograms show more and a broader spectrum of deviations for AnxA5 (Fig. 5d) than for [^18^F]FDG (Fig. 5c). Differences in the uptakes of the two tracers are probably more pronounced in Fig. 5c, d since the data from the entire tumour volume is used instead of the two dimensions only in Fig. 5a, b, e, f.

This last comparison of the uptakes of the widely differing tracers [^18^F]FDG and AnxA5 in the same tumour was also performed in more tumour-bearing mice and the effects of changing several post-processing parameters on the calculated HFs and their ratios were examined:

- Permuting the planes from *Z*- to *X*- or *Y*-axes and subsequently recalculating the whole VOI HFs changed the HF values in the same direction for both tracers and essentially to the same degree. Thus, the permutation had very small effects on the ratio of the HF of AnxA5 to that of [^18^F]FDG, indicating the algorithm handles calculation similarly for planes independent of the direction.
- When filtered back projection (FBP) was used instead of the default OSEM reconstruction, the calculated HFs decreased for all images, which is consistent with the less detail obtained with FBP than with iterative reconstructions such as OSEM. However, the HF for AnxA5 was still consistently higher than that of [^18^F]FDG.
- Increasing or decreasing the VOI volume by ≈20 % affects the number of pixels of the tumour included in the calculations. In some cases, the calculated HFs for the two tracers increased and in others they decreased, which might be indicative of some differences in distribution and/or edge phenomena between the two tracers. However, in all four comparisons, the HF for AnxA5 was always greater than for [^18^F]FDG.
- Using half the pixel size in the reconstruction but keeping other parameters constant can be seen as a form of smoothing which reduces the amount of detail in the image. Similar to using FBP, all the calculated HFs for all images decreased, but the HFs for AnxA5 were still consistently higher than for [^18^F]FDG.
- Using a larger lower limit (from a resolution of 1.2 (CFOV) to 2) as a cut-off when thresholding for the peaks accepted had the largest effect: The calculated HFs are, as expected, much smaller since they will be based on far fewer events. All HFs decreased and those for [^18^F]FDG decreased more than those for AnxA5, so the one-on-one comparison still gave the same result: that the AnxA5 images were more heterogeneous even when based on far fewer peaks.

## Discussion

Patterns of uptake in a region can be generally described by using maximum-, minimum- and mean uptakes and the ranges between these. These general parameters can be viewed as a type of ranking of the characteristics of the radioactivity uptake. Therefore, any region of interest that is delineated by the camera software can generate a four-column matrix, which gives something of a ranking of the heterogeneity in the image. Although these parameters are widely used, they are not descriptive of all the characteristics in a volume of interest. The algorithm developed here, on the other hand, calculates a HF that takes into account parameters such as the distributions of the uptake variations throughout the entire VOI, how frequent these variations are and how quickly or slowly these changes occur in an area of interest. To generate a more universally applicable algorithm, numerous loops were included so that modifications would not be required depending on the structure of the data.

Different ways of calculating the distances between uptake variations were examined. Originally, peaks were related to the centre of the VOI, which was defined to be the centre of the largest plane. However, this assumed that the tumour or, more generally, the tissue of interest was in essence a sphere, which in reality is seldom the case. It was therefore difficult to reliably find the centre in the analysed tissues. Thus, rather than defining and relating peaks to the centre, both A and B were instead defined to be peaks. The distances between A and B then had to be limited to predefined values in order to minimise size effects. Here, the distances were limited to be no less than the resolution of the camera so that unresolved peaks would not be included in the calculations and not more than the diameter of the smallest tumour dimension. Using different cut-off limits will, of course, directly change the number of peaks used in the HF calculations. In this study, the xenografts were placed close to the CFOV and this was therefore deemed to be a reasonable lower cut-off limit. When the xenografts are placed further from the CFOV (where the resolution can be much larger), a larger cut-off limit would be more appropriate. Changing to a larger cut-off (Table 1) did change the HFs but not the overall results of the one-on-one comparisons. In clinical imaging, tumours can be and are located throughout the body and the choice of the cut-off limits should be made with consideration for the resolution at that particular location in the FOV. It is also important when making comparisons between different imaging sessions to maintain similar placements in the camera to reduce the impact of differing resolutions on the results. In cases in which a deviation from the mean uptake has several peak values in close proximity to each other, it should only be counted as a single peak. By imposing a minimum distance restriction, deviations with this kind of topography will not be split into different contributions. Thus, the distances and the limits imposed restrict which peaks are included in calculations by the algorithm.

**Table 1:** Heterogeneity factor (HF) for the uptake of AnxA5 and [^18^F]FDG in FaDu xenografts in four mice, the ratios between their calculated HFs and the effects of altering post-scan processing parameters on these HFs and their ratios

Tracer	HF^a^	Alternate permutation^b^		Reconstruction^c^	Image size^d^	VOI volume change^e^		Cut-off limit^f^
		Coronal	Sagittal	FBP	256 × 256	≈ +20 %	≈ −20 %	→2 mm
AnxA5	0.1342	0.0545	0.0930	0.1004	0.0827	0.1620	0.1363	0.0287
[^18^F]FDG	0.1006	0.0377	0.0677	0.0869	0.0376	0.1334	0.0723	0.0157
Ratio^g^	1.334	1.446	1.374	1.155	2.199	1.214	1.885	1.828
AnxA5	0.3570	0.0892	0.1328	0.2881	0.1036	0.3354	0.1976	0.2351
[^18^F]FDG	0.3101	0.0751	0.1061	0.1911	0.0805	0.2710	0.1115	0.1480
Ratio^g^	1.151	1.188	1.252	1.508	1.287	1.238	1.772	1.589
AnxA5	0.2558	0.0724	0.0919	0.1694	0.0825	0.2856	0.2708	0.0944
[^18^F]FDG	0.1147	0.0310	0.0442	0.0982	0.0389	0.1879	0.0783	0.0209
Ratio^g^	2.230	2.335	2.079	1.725	2.121	1.520	3.458	4.518
AnxA5	0.3036	0.0701	0.0940	0.2870	0.0825	0.3701	0.2169	0.0966
[^18^F]FDG	0.1199	0.0255	0.0330	0.0955	0.0321	0.0991	0.0818	0.0292
Ratio^g^	2.532	2.749	2.848	3.005	2.578	3.735	2.652	3.308

^a^HF for VOIs drawn as in “Methods” (“VOI definition” section), axial (*Z*-axis) planes, OSEM2D reconstruction, 512 × 512 pixels and the lower cut-off for resolution at centre of field-of-view (FOV) at 1.2 mm

^b^Permutation for the planes in the VOI to be along the *X*- (coronal) or *Y*- (sagittal) axis instead of the *Z*-axis

^c^Filtered back projection reconstruction was used instead of OSEM2D

^d^The pixel size was dubbled

^e^The size of the VOI used in the HF calculations was increased or decreased by 20 %

^f^Lower cut-off limit changed from resolution at CFOV (1.2 mm) to that toward the outer edge of FOV (2.0 mm)

^g^Ratio = HF_AnxA5_/HF_FDG_

Simply calculating a number for the heterogeneity does not indicate which variations in the tissue contributed to the size of that number, which may be important when making inter- and intra-individual analyses. Histograms were therefore also created for each of the experimental situations (C and D in Figs. 3, 4 and 5) in order to visualise how the contributions to the HF were distributed in the tumour, how frequent they are and how quickly or slowly the changes are occurring. This type of information has been of interest when attempting to understand local variations in the underlying tumour biology (see e.g. [41]).

It has been shown previously (e.g. [31, 42, 43]) that heterogeneity determinations are affected by tumour volume. For example, partial volume effects (PVEs) can mask substantial uptake variations in small lesions. Influences of PVEs were minimised here since we only analysed tissues from tumours with dimensions that were at least fourfold that of the resolution of the camera [37]. When the volumes of the VOIs used were increased or decreased by ≈20 % here, the calculated HFs for AnxA5 and [^18^F]FDG did not change in a strictly parallel fashion. This could possibly be due to differences in regional tracer deposition patterns that were included or excluded by the changing VOI size. However, increasing the size of the VOI may include more edge pixels that will have a larger background contribution whereas decreasing the volumes may lead to under-sampling [42]. Therefore, the overall effects of changing VOI sizes can be difficult to predict. Another type of size effect was also observed when applying this algorithm. When peaks from multiple planes were included in the calculations, the HFs increased very rapidly in a non-linear fashion for each extra plane included. To curb the uncertainties introduced by these effects, the heterogeneity was instead analysed plane-wise and then added.

During the development of the algorithm, it was observed that relatively small variations in VOI delineations using the IRW thresholding tools could lead to substantial changes (up to approximately 30 %) in the calculated heterogeneity, even though the histograms were similar in appearance. When the VOI delineations were instead first performed using thresholding and then adjusted to conform to the dimensions determined postmortem, as described in the “Methods” section, the reproducibility in reanalyses of the HFs was better than 98 %. This method was therefore used in all the subsequent analyses. The camera used here was a stand-alone PET instrument. Using the morphological information from a combined PET-MRI or PET-CT would be expected to also reduce uncertainties due to VOI delineations.

Three examples of comparisons were analysed here and, for the first two, only one animal/tumour was used for each tracer. This is naturally not a large enough sample size for drawing statistically sound conclusions about differences between individuals (e.g. [44]) nor was that the goal here. These cases were chosen as illustrations to demonstrate whether this heterogeneity algorithm could handle these types of comparisons, which typically occur in preclinical studies. During the development of the algorithm, multiple additional matrices that were shorter but had the same mathematical appearance as the biologically generated matrices were also manually constructed. This allowed us to confirm in a controlled manner how small alterations of the algorithm handled a particular problem. Then, in the final wider application, the HFs of [^18^F]FDG and AnxA5 in four tumour-bearing animals were calculated for different post-scan processing parameters. This demonstrated that, while the values of the calculated HFs changed sometimes quite a lot, the relative heterogeneity difference between the two tracers (i.e. AnxA5 more heterogeneous than [^18^F]FDG) held for all animals and all processing parameters tested. Future applications should apply the algorithm in larger sample sizes of even more varied populations.

Addressing the influence of the gradient proved to be somewhat problematic. Initially, this was attempted by including a fifth column containing the gradient. However, this column would need to have a different structure than the original matrix, since the gradient would be composed of two figures describing the rise and the fall of the peak. This would inevitably be rejected, since a prerequisite for the calculations is that columns are the same size and form. Since our aim was to generate a code that would not require modification depending on the data, an alternative method that allowed the original matrix characteristics to be maintained had to be developed. We decided instead to use the indirect approach, in which the shape of the gradient was used as a criterion for when a peak was accepted or rejected, as described in “Recreation of a cell array with individual intensities related to the mean of the associated plane” section. The impacts of indirect approaches are usually less straightforward to control or maintain, but the treatment of the gradient effects was considerably simplified by this approach. Thus, when exercising control over the behaviour of the HF, each plane was calculated separately in order to decrease the impact of the non-linear effect of size and also to incorporate the effect of the rise and fall of the slope. As a consequence, the column that contains the different *Z* locations was omitted. Information from a single peak can now give different contributions in different planes, the impact of which needs to be addressed in future developments.

The methods most often employed to examine heterogeneity in PET or SPECT imaging data usually assess only pre-determined cross-sections [16]. Restricting the calculations to only certain planes assumes that the biology and image features (noise, etc.) have the same characteristics in each plane, which is not necessarily the case. Nor does it take intra-tumour variability into account. El Naqa et al. performed a one-dimensional assessment of uptake variation by forming what they called an intensity-volume histogram [21]. This approach, similar to the dose-volume histograms employed in radiation therapy, was however a simplified one, since several parameters that influence heterogeneity had to be omitted in the one-dimensional assessment. In the algorithm presented here, the entire tumour volume was analysed. This unfortunately introduced a considerable non-linear size dependency in inter-tumour comparisons. When a linear correction normalising the HFs by dividing by the original matrix size was included, the size dependency decreased considerably but not completely. Further developments should attempt to improve the treatment of the non-linearity of the size dependency or investigate the possibility of stratifying into similarly sized tumours [43].

The general uptake level estimated with e.g. SUV is one of the parameters most often used for ranking and describing the radioactivity uptake in a tissue. Tissues with large general uptakes would have higher HFs than tissues with low general uptakes since the |*I*_*p*_ − *Ī*_*k*_| would automatically be larger. To attempt to compensate for the impact of uptake sizes on the factors calculated, the uptake deviations were normalised to the maximum uptake deviation in each plane. Thus, they were all kept between 0 and 0.83 (the *X-*axis of the histograms). It should be noted that the smaller the deviation, the lower the probability that they will lead to deviations subtracted from the mean (the *X-*axis). In this example, there are fewer contributions near zero (and also near 0.83) in the histograms. Therefore, calculations of the HF and the associated histograms can potentially provide additional information about the textural features contributing to a general uptake level estimation.

Biological explanations of the differences behind the different HFs calculated for the examples presented here may be difficult to make [6] and are beyond the scope of this article. However, some general comments may be made.

In example I, the heterogeneity of the uptake of the control mTrx-GFP tracer was larger than that of the AnxA5-based tracer. The uptakes analysed here were SUVs and not macroparameters (such as binding potentials) separating the specifically from non-specifically bound tracer. It has been previously discussed [23] that the control protein may visualise enhanced permeability and retention (EPR) [40] effects while the AnxA5 tracer uptake may be due to both specifically bound tracer as well as EPR contributions. The AnxA5 tracer appears to be more homogenously distributed into the tissue while there is an increased frequency and a broader spectrum of uptake deviations in the mTrx-GFP (Fig. 3c, d). It could be of interest to investigate in a broader study whether such observations can be further correlated to e.g. immunohistochemical analyses of morphology and target expression levels that affect their uptakes to bridge the resolution gap between histochemical and in vivo analyses.

In example II, the uptakes of a targeting Affibody™ tracer in two different xenografts in two animals were compared. It is very apparent from the transaxial images (and the surface plots) that the total uptake in A431 is very much smaller but also has a more irregular distribution throughout the tissue than in SKOV-3. Even though the two tumours may differ in their microenvironmental characteristics, the large differences in uptake patterns have been primarily attributed to the tracer targeting capability since SKOV-3 has substantially larger HER2 expression levels than A431. The differences between the two uptakes have therefore been interpreted to reflect specific uptake [35], where specific uptake in SKOV-3 is to a large degree equal or more evenly distributed throughout the VOI. The increased HF for A431 is also (from looking at Fig. 4c, d) much more frequent and more evenly distributed over the entire *X*-axis of the histograms.

In example III, the uptakes of two quite different tracers in the same xenograft and animal were examined on the same day and with the same imaging parameters. The HF for [^18^F]FDG in this model was considerably smaller than that for AnxA5. This could be due to the fact that [^18^F]FDG, due to its much smaller size, should be able to more readily diffuse into the tumour and therefore be more evenly distributed. However, these textural features might also indicate regional differences in tracer retention due to increased metabolic demand from that due to cell death, which would be interesting to examine further in future studies.

Comparisons of heterogeneity, as evaluated in this current version of the algorithm, should be made on a one-to-one basis and not group-wise, since individual parameters still have a substantial impact on how the HFs are calculated. For example, in example I and III, the spherical VOIs used were constructed differently, in order to include as many parameters as possible between the tissues being compared. Therefore, for a comparison of AnxA5 in the two different animals in I and III, a new analysis should currently be performed after adjustments of the VOI delineations for that particular comparison. Otherwise, the algorithm will have to be adjusted so that nonlinear corrections for size effects can be made.

As discussed elsewhere [45], heterogeneity must be handled nonlinearly, i.e. the parameters will not be modified in the same manner continuously. Each characteristic that was handled by the algorithm (distances between deviating peaks, the gradients and the size compensations) also had differing, nonlinear impacts on the calculated heterogeneity. These nonlinear impacts are difficult to handle in a rigorous fashion. In order to make this heterogeneity algorithm more generally applicable, we have here built in methods that attempt to handle, albeit in a linear fashion, these three contributions.

While developing and validating this algorithm, we have attempted to initially control as many pre- and post-processing parameters as possible while varying only one at a time. In some ways, this is more easily achieved in preclinical than in clinical imaging, although each has its own advantages and disadvantages. The variability of many imaging aspects that affect quantification (e.g. [6, 38]) must also be considered and standardised in wider applications of this method to estimate heterogeneity. For example, uptake intensity variations can be a reflection of the inherently noisy image accusation of PET, or PVEs [46]. Thus, these influences on uptake variations need to be addressed, even though stronger signals usually observed in tumours will increase the signal-to-noise ratio. Systematic errors that influence heterogeneity can be present in each comparison and may not influence the conclusions drawn at one site when the comparisons are made as ratios of one image to the other. Therefore, inter-site comparisons will probably contain non-comparable systematic error influences, such as differences in minimum performance standards. Future work toward a wider utilisation of this algorithm should therefore also examine the system-specific influences on the comparisons made. Increasing the number of iterations in iterative OSEM or MAP reconstructions will affect the heterogeneities [47]. Changing the reconstruction algorithm from OSEM to FBP here affected the amount of detail but did not change the overall result of which tracer uptake was more heterogeneous. The pixel size used in the reconstruction is important for the final size of the HF, since dividing the image into a different number of picture elements would directly affect the number of peaks. Since this method attempts to determine heterogeneity of the whole volumes instead of single planes, permuting the direction of planes when calculating the HF may reveal direction-related biases and is therefore recommended as a complimentary analysis. Standardising the post-processing might not always be possible and, as shown in the comparisons in Table 1, perceived differences in comparisons could in fact be due to the different processing parameters used. Future inter- as well as intra-site applications of this algorithm should either standardise these parameters when performing comparisons between individuals or analyse the effects of these parameters on the HFs calculated.

## Conclusions

An algorithm was developed here that could analyse the heterogeneity of radioactivity uptake in small animal PET images. It was constructed to assess the entire tissue volumes instead of solely a single cross-section. It has built-in strategies for dealing with different image features that might skew the calculated heterogeneity inappropriately. Each contributing parameter had different effects on the heterogeneity and therefore strategies for handling these parameters had to be separately developed and optimised. The histograms may be a valuable complement for visualising how the contributions to the heterogeneity are distributed within an entire tissue volume. Wider future applications may require some modifications to specifically address larger inter-group variations. At this stage, the algorithm is rather robust for one-on-one comparisons of similar volume preclinical tumours.
